# Pulse-wave velocity is associated with cognitive impairment in haemodialysis patients

**DOI:** 10.1042/CS20170973

**Published:** 2017-06-28

**Authors:** Eve Miller-Hodges, Neeraj Dhaun

**Affiliations:** University/British Heart Foundation Centre of Research Excellence, University of Edinburgh, The Queen's Medical Research Institute and Department of Renal Medicine, Royal Infirmary of Edinburgh, Little France Crescent, Edinburgh, U.K.

**Keywords:** arterial stiffness, cognitive impairment, chronic kidney disease

## Abstract

Chronic kidney disease (CKD) is common, its prevalence increasing with age. Cognitive impairment is common in the elderly, in CKD and in those on maintenance haemodialysis. As cognitive impairment is the precursor to dementia, the identification of reversible risk factors for cognitive decline is the key to reducing dementia risk. Arterial stiffness is one such potential risk factor. It is independently associated with cardiovascular outcome in dialysis patients. Importantly, the recent demonstration of an independent association between arterial stiffness and cognitive impairment in these patients suggests that vascular stiffness might be potentially causative in the development of cognitive impairment and also be an opportune target for interventions. Whether unstiffening of blood vessels in patients on maintenance haemodialysis can reduce the incidence of cognitive impairment or indeed slow its progression to dementia, remain unanswered questions. In this issue of the *Clinical Science*, Angermann and colleagues present thought-provoking data related to cognitive impairment in haemodialysis patients.

## Arterial stiffness and cognitive impairment in chronic kidney disease

Chronic kidney disease (CKD) is common, affecting more than 10% of the global population [1]. Its prevalence increases with age and approximately half of people over the age of 70 have CKD [1]. The impact of an aging population is that more older people with CKD are progressing to end-stage renal disease, requiring dialysis. As glomerular filtration rate (GFR) declines, the risks associated with CKD increase. These include cardiovascular disease, which is the commonest outcome of CKD [1], and cognitive impairment [2].

Cognitive impairment is defined as a disproportionate decline in cognition than that expected with normal aging. A continued ability to undertake activities of normal daily living discriminates it from dementia. The prevalence of cognitive impairment increases with age, but it is also a major problem in patients receiving maintenance dialysis, with rates as high as 70% [3]. As the average age of patients starting dialysis in the U.K. is approximately 65 years [4], this represents a significant healthcare burden. Importantly, as cognitive impairment represents a transitional phase between normal function and the development of dementia, it offers a potential opportunity for therapeutic intervention. The identification of reversible risk factors that might contribute to cognitive decline is the key to reducing dementia risk.

Cognitive impairment affects a patient’s ability to retain new information, their decision-making skills and their adherence to often demanding medication and lifestyle regimens [5]. Cognitive impairment is also associated with poorer outcomes for those on dialysis [6] These are even worse when there is co-existing dementia with a 2-year survival rate of 20% compared with approximately 70% in patients without dementia [7]. A recent meta-analysis of more than 50000 patients with *predialysis* CKD showed that these patients have poorer cognition than those with normal renal function [8]. Transition to dialysis appears to increase the rate of cognitive decline [9]. This mirrors the rapid increase in cardiovascular risk seen once patients start dialysis [10].

In this issue of the *Clinical Science*, Angermann and colleagues present thought-provoking data related to cognitive impairment in haemodialysis patients [11]. In a cross-sectional observational study, they performed cognitive testing in approximately 200 self-selected patients. Their haemodynamic assessments included measures of arterial stiffness using the gold standard of pulse-wave velocity (PWV). Both epidemiological and clinical data show that damage to large arteries contributes to the increased cardiovascular risk observed in CKD [12]. Arterial stiffness causes an elevation in central systolic blood pressure, increasing left ventricular workload with the gradual development of left ventricular hypertrophy, and also causes a fall in diastolic blood pressure, impairing coronary blood flow. It is an important independent predictor of all-cause and cardiovascular mortality in patients with CKD and it progresses most rapidly in patients on dialysis [13,14]. In the current study, the authors demonstrate an independent relationship between cognitive impairment and arterial stiffness in this patient group.

Using the Montreal Cognitive Assessment (MoCA)–a sensitive and specific tool for detecting mild cognitive impairment–the authors found that more than 60% of patients studied had cognitive impairment. The greatest deficits were seen in executive function, verbal fluency and memory recall, all areas most likely to impact upon patient education and decision-making. As expected, those with cognitive impairment were older, had a lower educational level and higher rates of overt cardiovascular disease. Interestingly, dialysis vintage and dialysis adequacy did not differ between the two groups. PWV was significantly higher in those with cognitive impairment than in those without it (10.3 m/s compared with 8.1 m/s). Following adjustment for traditional risk factors, PWV had a stronger association with cognitive impairment than even age. The threshold of 9.8 m/s generated in this cohort is consistent with the threshold for increased cardiovascular risk of 10.0 m/s recommended by the *European Society of Hypertension* [15], suggesting good generalizability.

A number of studies have demonstrated that a higher PWV (and/or carotid artery pulsatility, as another measure of arterial stiffness) is associated with structural brain lesions such as white matter hyperintensities and cortical infarcts, indicative of microvascular damage [16–18]. This supports arterial stiffness as a potential surrogate measure of the brain microcirculation [19]. In the general population, arterial stiffness is strongly associated with both cognitive impairment and cognitive decline [20]. Crucially, this relationship is confirmed by longitudinal studies, supporting a causative role [21].

Although limited by its cross-sectional design and relatively small patient numbers, the current study raises the question as to whether increased arterial stiffness might partly explain the higher rates of cognitive impairment and aggravated cognitive decline seen in dialysis patients. Noteworthy is that the patients included were self-selected, increasing the chance of recruitment bias, an inherent problem in such studies [22]. Furthermore, patients were assessed immediately prior to a dialysis session. This is to be recognised when patients perform worst [23] and so the current results might have been less clear-cut had the assessments been performed postdialysis. The MoCA was the single measure of cognitive function. Although it has been validated in dialysis patients [24], it might not capture the overall burden of cognitive impairment. Nevertheless, similar findings have been reported in a smaller study which used more comprehensive cognitive testing [25]. Detailed cognitive assessment is time-consuming, but dialysis patients present a unique cohort with prolonged, set times for hospital attendance, making this feasible.

The current study poses several intriguing questions. First, are those with stiffer blood vessels more likely to develop cognitive impairment (and potentially dementia)? Second, can reducing arterial stiffness slow cognitive decline in dialysis, and more broadly, CKD patients? Standard therapies that reduce arterial stiffness in other settings might be less effective in end-stage renal disease. For example, hypertension in those on dialysis is mainly influenced by fluid removal as opposed to pharmacological management. There also exist other mechanisms to reduce arterial stiffness independent of blood pressure; these include the use of phosphate binders such as sevelamer [26]. Perhaps more frequent dialysis might offer another solution. Certainly, a few small studies have shown an improvement in cognition following renal transplantation when GFR increases rapidly [27,28]. This even included improvements in white matter integrity on brain imaging [29]. Whether these changes are related to the unstiffening of blood vessels seen with transplantation [30] is unclear.

Disentangling the complex interplay between arterial stiffness, chronic kidney and cardiovascular disease and cognitive function will be crucial in identifying those patients most at the risk of cognitive impairment. Novel imaging techniques such as optical coherence tomography of the eye enable direct, rapid, noninvasive imaging of the eye’s microvasculature. This could offer a surrogate measure of the systemic microvascular disease burden affecting both the brain and kidney [31]. Biomarkers associated with increased cardiovascular risk, such as high-sensitivity cardiac troponin, might also identify those patients most at the risk of cerebral microvascular disease and therefore cognitive impairment [32]. These may both then be utilised in interventional studies using agents such as endothelin-receptor antagonists, which have the potential to reduce arterial stiffness and so cognitive impairment [33]. Most importantly, larger, prospective and longitudinal studies are urgently needed to address whether reducing arterial stiffness can halt the decline in cognitive function seen in patients with CKD, a group at extremely high cardiovascular and dementia risk.

## Abbreviations

CKD: chronic kidney disease
MoCA: Montreal Cognitive Assessment
PWV: pulse-wave velocity
